# How Time Attitudes Shape Academic Success: The Protective Pathways of Emotion Regulation and School Well-Being in Adolescents

**DOI:** 10.3390/jintelligence14060095

**Published:** 2026-06-01

**Authors:** Yosselyn Valdebenito-Montecino, Claudio Bustos, Yaranay López-Angulo, Úrsula Cea-Monsalves, Javier Latorre-Nanjarí, Wenceslao Peñate, Frank C. Worrell, Cristian Oyanadel

**Affiliations:** 1 Department of Psychology, Faculty of Social Sciences, Universidad de Concepción, Concepción 4030000, Chile; yossevaldebenito@udec.cl (Y.V.-M.); clbustos@udec.cl (C.B.); yaralopez@udec.cl (Y.L.-A.); urscea@udec.cl (Ú.C.-M.); 2 Department of Psychology, Universidad de La Serena, La Serena 1700000, Chile; jlatorren@userena.cl; 3 Clinical Psychology, Psychobiology and Methodology School, Psychology Faculty, Guajara Campus, Universidad de La Laguna, San Cristóbal de La Laguna, 38200 La Laguna, Spain; wpenate@ull.edu.es; 4 Berkeley School of Education, University of California, Berkeley, CA 94720-1670, USA; frankc@berkeley.edu

**Keywords:** time attitude profile, academic achievement, mediation analysis, school well-being, emotion regulation, adolescence

## Abstract

Time perspectives represent a fundamental dimension of adolescent development that may influence academic outcomes through emotional and interpersonal mechanisms. In this study, we examined how time attitude profiles relate to academic achievement in language and mathematics and whether emotion regulation difficulties and school well-being function as mediating mechanisms. A sample of 257 Chilean adolescents with a mean age of 14.36 years (*SD* = 0.57) from nine schools completed self-report measures of time attitudes, emotion regulation difficulties, and school well-being and provided school grades in language and mathematics. Latent profile analysis identified three time attitude profiles: Ambivalent, Positive, and Optimistic. Descriptive analyses showed that students with Positive and Optimistic profiles reported higher school well-being and fewer emotion regulation difficulties than Ambivalents, although mean differences in academic achievement across profiles were small. In mixed-effects models, time attitude profiles did not robustly predict language achievement once covariates were controlled, and only adolescents with an Optimistic profile showed slightly higher mathematics achievement than Ambivalents. Mediation models indicated that school well-being significantly mediated the association between the Positive time attitude profile and both mathematics and language achievement, whereas emotion regulation difficulties did not emerge as significant mediators for either subject. These findings highlight school well-being as a key psychosocial mechanism linking positive temporal orientations to academic outcomes and suggest that school-based interventions targeting time attitudes and school well-being may foster adolescents’ academic success.

## 1. Introduction

Adolescence represents a transitional phase of development characterized by accelerated physical, cognitive, emotional, and social transformations. This developmental period generally spans approximately 10 to 19 years of age, marking the bridge between childhood dependence and adult autonomy (Organización Mundial de la Salud, 2018). Early and middle adolescence constitute critical stages for the consolidation of self-regulatory capacities. During this developmental period, the neurobiological systems underlying voluntary control of attention and affective responses undergo substantial maturation and reorganization. The prefrontal cortex, which is involved in executive functions such as impulse inhibition and emotional modulation, undergoes significant dendritic pruning and myelination during adolescence—processes that enhance neural efficiency while simultaneously generating temporary vulnerabilities in regulatory capacities (Kaunhoven & Dorjee, 2017). Consequently, managing the convergence of heightened emotional sensitivity, emerging social pressures, and still-developing regulatory systems constitutes one of the defining challenges of adolescence (Fombouchet et al., 2023).

In this context, it has been proposed that time profiles influence cognitive–emotional self-regulation processes, as they shape individuals’ interpretations of events and themselves (Stolarski et al., 2020). Individuals with balanced time profiles tend to use their temporal flexibility to regulate emotions and mood states effectively (Stolarski et al., 2014). Likewise, positive profiles are associated with higher levels of subjective well-being (Burzynska-Tatjewska et al., 2022). These outcomes are particularly relevant in educational settings, where positive time attitudes toward the future act as protective factors for academic performance (Hao et al., 2024). Y. Li (2025) posited that although such attitudes predict academic achievement positively, this relationship remains underexplored, with potential moderating variables yet to be identified.

In this study, we understand time perspective as a cognitive-motivational construct encompassing individuals’ thoughts and feelings about the past, present, and future (Mello & Worrell, 2015; Zimbardo & Boyd, 1999). Within this framework, we specifically focus on time attitudes, defined as the affective evaluations (positive or negative) toward each temporal period, which have shown particular relevance in adolescence for explaining academic outcomes and well-being. Throughout the manuscript, we use time perspective to refer to the general construct and time attitudes when discussing the specific affective dimension assessed in this study.

Therefore, understanding the interplay among individual differences in time perspective, emotional regulation capacity, and subjective satisfaction with the school environment may reveal protective mechanisms that foster academic success during this highly sensitive developmental period. Building on this premise, the present study aims to determine whether positive temporal attitude profiles influence academic performance not only directly but also indirectly through school well-being and emotional regulation, which function as key mediating variables.

### 1.1. Emotion Regulation: Conceptual Framework and Academic Relevance

Emotion regulation encompasses the dynamic processes through which individuals modulate the initiation, intensity, duration, and expression of emotional responses across varying situational contexts. Rather than conceptualizing emotion regulation as a unitary construct, contemporary models recognize it as a multifaceted system involving awareness of one’s emotional state, acceptance of emotions as valid experiences, strategic inhibition of counterproductive behavioral impulses during periods of heightened affect, and deployment of cognitively and behaviorally adaptive coping strategies calibrated to situational demands (Gratz & Roemer, 2004; Tang et al., 2015).

The developmental trajectory of emotion regulation during the adolescent transition reveals a complex pattern. Although many adolescents progressively strengthen their affective management competencies through accumulated experience and enhanced cognitive capacity, others experience deterioration in regulatory function, manifesting in clinical presentations of mood and anxiety disorders (Silvers, 2022). Neuroimaging research comparing adolescent and adult samples reveals a salient pattern: adolescents demonstrate significantly greater amygdala reactivity—the brain region associated with emotional salience detection—coupled with relatively diminished prefrontal engagement during effortful emotion regulation attempts. This neurobiological dissociation between heightened emotional sensitivity and still-maturing regulatory control systems substantially reduces the effectiveness of deliberate emotion management strategies (Stephanou et al., 2016).

Within educational settings, the regulatory demands are substantial and continuous. Academic learning requires sustained attentional focus, tolerance for performance errors and corrective feedback, and persistence through challenging material—all domains where emotional interference can substantially compromise performance. Research demonstrates that students exhibiting more sophisticated emotion regulation capacities consistently achieve higher academic outcomes, partially because such competencies permit more efficient allocation of cognitive resources to learning tasks rather than to managing disruptive emotional states (Harley et al., 2019; Khan & Jameel, 2024; Usán & Quílez, 2021). Longitudinal evidence indicates that when adolescents cultivate positive emotional states through effective regulation, these affectively positive conditions generate reciprocal benefits for academic motivation and achievement (Pekrun et al., 2017).

### 1.2. School Well-Being: Conceptual Framework and Academic Relevance

School well-being integrates cognitive appraisal processes—individuals’ evaluative judgments about their school experiences—alongside the phenomenological manifestations of those evaluations across emotional, physical, and social registers. Definitions in the extant literature vary considerably, though converging evidence suggests that school well-being emerges when students perceive their individual psychological and developmental needs as adequately satisfied within the academic context, when they experience predominantly positive rather than negative emotional states at school, and when they perceive themselves as functioning effectively across academic, social, and personal-developmental domains (Hossain et al., 2023; Obermeier et al., 2021).

In this regard, school well-being comprises both cognitive and affective components. The cognitive component involves students’ subjective evaluation of their school life across multiple domains (e.g., teacher–student relationships, learning experiences), and the affective component reflects the balance between positive and negative emotions experienced within the school environment (Benavente et al., 2018). Tian et al. (2014) proposed a tripartite model in which school well-being encompasses school satisfaction, positive affect, and negative affect. Positive affect refers to the pleasant emotions a student experiences at school, whereas negative affect represents the unpleasant emotions that may arise from the school context.

Antecedent conditions fostering school well-being operate at multiple ecological levels: classroom and institutional factors such as instructional quality, teacher interpersonal warmth, and equitable classroom management systems; peer relationships characterized by reciprocal support and relative freedom from peer victimization; and individual dispositional factors including learning-oriented motivation and emotional resilience (Wentzel, 2024). The social relational dimension warrants particular emphasis, as students who experience relative isolation, conflict with peers, or strained relationships with teachers consistently report diminished school well-being levels, whereas students embedded in supportive social networks within the school environment demonstrate enhanced psychological adjustment (Saxer et al., 2025).

A recent meta-analytic synthesis reveals a consistently positive association between school well-being and academic performance, though the magnitude of this relationship proves modest in effect size (Kaya & Erdem, 2021). One proposed mechanism involves enhanced school engagement: when students experience the school environment as satisfying and supportive, they demonstrate heightened behavioral and cognitive engagement with academic tasks, which in turn facilitates achievement gains (Schnell et al., 2025). Comparative analyses of ecological predictors of academic achievement indicate that school climate—the collective social-psychological atmosphere—exhibits stronger predictive utility for achievement outcomes than classroom-level climate variables alone, reflecting the importance of broader institutional factors (Erdem & Kaya, 2023). Given that adolescents allocate substantial proportions of their waking hours within school environments, the identification of individual difference factors that contribute to greater school satisfaction becomes a matter of significant developmental and educational importance (Steinmayr et al., 2018).

### 1.3. Time Perspective: Conceptual Framework and Academic Relevance

Time perspective constitutes a metacognitive-affective process through which individuals categorize and frame their experiences along temporal dimensions. This categorical framing confers coherence, sequential ordering, and existential meaning to otherwise disparate experiences. Time perspective operates largely outside conscious awareness, yet profoundly shapes how individuals prioritize activities, evaluate life circumstances, project themselves into imagined futures, and ultimately construct their life narratives (Zimbardo & Boyd, 1999).

One prominent theory proposes that individual differences in time perspective cluster along five primary dimensions (Zimbardo & Boyd, 1999). Past positive reflects favorable evaluative perspectives toward personal history—a tendency to view previous life experiences and memories with satisfaction and nostalgia. Past negative represents a countervailing orientation emphasizing aversive historical experiences, whether through persistent rumination on previous difficulties or pervasive negative reappraisal of past events. Present hedonistic characterizes an orientation prioritizing immediate gratification, risk-taking, and sensory pleasure-seeking in the current moment. Present fatalistic denotes a resignation toward present circumstances, characterized by perceived helplessness regarding current events and pessimistic appraisals of proximal outcomes. Finally, the Future dimension encompasses goal-directed planning, delay of gratification, and structured anticipation of long-term consequences, reflecting individuals’ capacity to envision and work toward distant aspirations (Zimbardo & Boyd, 2009).

Individuals typically demonstrate differential emphasis across these temporal dimensions. Such imbalanced time perspectives frequently produce adaptive costs. For instance, excessive future orientation can generate tunnel vision around distant objectives, precipitating social isolation and diminished present-moment satisfaction. Conversely, pronounced past-negative orientation traps individuals in repetitive rumination on irreversible events, constraining their capacity to engage adaptively with current circumstances (Boniwell & Zimbardo, 2004).

In contrast, a Balanced Time Perspective (BTP)—characterized by cognitive and affective flexibility to shift among temporal frames according to situational affordances and developmental demands—represents a more psychologically adaptive stance. The balanced profile comprises strong positive past orientation (generating historical continuity and meaning), minimal negative past focus (avoiding maladaptive rumination), moderate hedonistic present engagement (permitting current satisfaction without recklessness), minimal present fatalism (supporting agency and hope), and robust future goal-directedness (enabling purposeful action and planning) (Zimbardo & Boyd, 2009).

Mello and Worrell’s (2015) research demonstrated that adolescents possessing optimistic time perspectives, characterized by favorable attitudes toward the future coupled with constructive views of the past, achieve measurably superior academic outcomes. This pioneering work established time attitudes as a significant individual difference variable with direct implications for educational success. Subsequent research has expanded upon this framework, revealing that positive past orientation, moderate present hedonism, and future goal-focus function as significant predictors of elevated subjective well-being in adolescent populations. Adolescents who maintain nostalgic yet realistic perspectives on past experiences, demonstrate moderate risk-taking and pleasure-seeking behaviors balanced against responsible decision-making, and maintain clear aspirations toward future accomplishments consistently report elevated positive affect, life satisfaction, and academic motivation (Kuan & Zhang, 2022).

Furthermore, future-directed time perspectives—characterized by optimistic yet grounded appraisals of possibilities ahead, combined with realistic confidence in one’s ability to influence outcomes through purposeful action and planning—contribute substantially to adolescents’ overall life satisfaction, psychological resilience, and educational persistence (X. Li et al., 2023). This future-oriented dimension appears particularly consequential in academic contexts, where the capacity to link present efforts to delayed gratification and long-term goals constitutes a fundamental requirement for sustained achievement.

Conversely, maladaptive time attitude profiles—featuring pervasive past-negative rumination, combined with hedonistic and fatalistic present orientations, and diminished future investment—correlate robustly with greater emotion regulation difficulties in adolescent samples. Such time attitude profiles may reflect or engender reduced capacity for emotional management, as indicated by research demonstrating that deviation from balanced time perspective is associated with elevated anxiety and depressive symptomatology, suggesting that temporal imbalance and affective dysregulation may constitute mutually reinforcing processes (Abdollahpour Ranjbar et al., 2025).

Recent research has shown significant associations between future-oriented time perspectives and enhanced academic self-efficacy, sustained motivational engagement, and improved academic performance. Adolescents with robust future goals demonstrate elevated confidence in their academic capabilities, heightened concern regarding the long-term consequences of their behavioral choices, and greater persistence when confronted with academic challenges (Marín-Velazco et al., 2022). Time perspective dimensions predict academic achievement outcomes, with particular strength evident for future orientation, positive past perspective, and balanced overall time attitude profiles (Boniwell & Zimbardo, 2004; Mello & Worrell, 2015). Longitudinal investigations demonstrate that future time perspective exerts both direct influences on academic achievement and indirect effects mediated through reductions in academic burnout and enhancement of academic engagement (Hong, 2025), supporting an integrated model wherein time perspective operates as a fundamental individual difference factor shaping multiple achievement-relevant outcomes. Furthermore, adolescent time attitude profiles, which integrate different temporal attitudes, have shown a significant relationship with academic performance and other socio-emotional variables (Andretta & Worrell, 2022).

In the current study, we investigated the protective pathways through which positive temporal attitudes promote academic achievement via enhanced emotion regulation and school well-being by examining mediation pathways. We hypothesized that (1) time attitude profiles, particularly those characterized by optimistic and positive attitudes, would directly predict academic achievement in both language and mathematics; (2) these favorable time attitude profiles would be associated with reduced emotion regulation difficulties and elevated school well-being; and (3) school well-being and emotion regulation would function as mediating mechanisms through which time perspective is associated with academic achievement. The inclusion of both language and mathematics achievement permits examination of whether proposed mechanisms operate uniformly across academic domains or demonstrate domain-specific patterns.

## 2. Materials and Methods

This investigation employed a retrospective ex post facto single-group design with a cross-sectional approach. Time attitude profile was considered a predictor variable, school well-being and emotional regulation were mediator variables, and language and mathematics achievement were outcome variables.

### 2.1. Participants and Sampling

This study was conducted across 9 subsidized educational institutions with similar socioeconomic indices, according to each school’s official vulnerability index, in Chile. At each participating institution, parental consent and student assent procedures were completed. The final sample comprised 257 adolescents attending secondary education, including 130 males (50.4%) and 128 females (49.6%), with a mean age of 14.36 years (*SD* = 0.57). This study received approval from the Ethics and Bioethics Committee at the Universidad de Concepción (protocol code: CEBB 1411-2023).

### 2.2. Instruments

Grade point average (GPA) in Mathematics and Language and Communication courses. Grades were obtained from the institution’s official records. These specific subjects were selected to avoid potential grade inflation in practical or sports-related courses, thereby providing a more accurate indicator of core academic competencies, in line with the domains assessed by Chile’s National System for the Measurement of Educational Quality (SIMCE).

Adolescent and Adult Time Inventory–Time Attitudes (AATI-TA). The Rio de la Plata Spanish version validated by Vásquez-Echeverría et al. (2020) was employed. This 30-item instrument includes six subscales: Positive Present (“I am happy with my current life”; α = 0.88), Negative Present (“I am not satisfied with my life right now”; α = 0.80), Positive Past (“I have very happy memories of my childhood”; α = 0.87), Negative Past (“My past is a part of my life I would like to forget”; α = 0.83), Positive Future (“I am optimistic about my future”; α = 0.88), and Negative Future (“I doubt that I will accomplish anything in life”; α = 0.76). Responses are given on a 5-point Likert scale ranging from 1 (Strongly disagree) to 5 (Strongly agree). Therefore, the reliability estimates for this sample ranged from α = 0.76 to α = 0.88.

Difficulties in Emotion Regulation Scale (DERS-E). The version adapted to the Chilean context by Guzmán-González et al. (2014) is a 25-item Likert-type scale assessing difficulties in emotional regulation, scored from 1 (Almost never) to 5 (Almost always). It comprises five subscales and yields a total score, with higher scores indicating greater difficulties in emotional regulation. The subscales are (1) Emotional rejection, reflecting difficulty accepting distress (“When I feel bad, I get angry with myself for feeling that way”; α = 0.90); (2) Emotional dyscontrol, referring to difficulty controlling behavior when experiencing distress (“When I feel bad, I lose control”; α = 0.90); (3) Emotional interference, indicating difficulty concentrating while experiencing negative emotions (“When I feel bad, I have difficulty completing tasks”; α = 0.85); (4) Lack of emotional attention, assessing low ability to attend to and understand emotions (“When I feel bad, I recognize my emotions”; α = 0.83); and (5) Emotional confusion, referring to difficulty identifying and clarifying emotions (“I get confused about what I am feeling”; α = 0.76).

Brief Adolescent School Well-Being Scale (BASWBSS). The Chilean version developed by Benavente et al. (2018) includes two subscales. The first consists of seven Likert-type items with six response options ranging from 1 (Strongly disagree) to 6 (Strongly agree), assessing the cognitive evaluation of school satisfaction (e.g., “I am doing well at my school”). The second subscale comprises two Likert-type items with four response options ranging from 1 (Never) to 4 (Always), measuring the affective component of school well-being (e.g., “I feel good at my school”) (Benavente et al., 2018). In the present study, only the cognitive component was analyzed (α = 0.78).

### 2.3. Procedure

Evaluations were conducted in person at each educational institution during a single session. To ensure anonymity and confidentiality, as stipulated in the informed consent and assent forms, no personal identifiers were recorded. Instead, responses were coded using identification numbers managed exclusively by the research team. Students had 45 min to complete all instruments during the time designated by each school. No incentives were provided for participation.

Analyses were performed using R software (Version 4.3.2). Prior to the main analyses, the pattern of missing data was examined. Given that some variables had missing values, multiple imputation was used as a strategy for their treatment, generating 15 imputed databases. Subsequent analyses were carried out using statistical methods adapted to imputed data.

First, students’ time attitude profiles were determined through latent profile analysis (LPA), identifying three distinct groups: Ambivalent, Positive, and Optimistic time attitude profiles. The LPA approach allows for the identification of individuals who share similar patterns across multiple temporal dimensions. To determine the specific structure of the variance-covariance matrix of the latent class profile model, the 14 covariance models proposed by Banfield and Raftery (1993) were analysed using the mclust package, testing solutions ranging from 3 to 9 categories. The model with the lowest BIC was selected, which prioritizes parsimonious solutions that achieve an adequate fit to the data. Additionally, the decision was guided by the theoretical interpretability of the solutions obtained, in accordance with the recommendations of Jung and Wickrama (2008).

Subsequently, descriptive statistics and comparisons were calculated by profile to examine differences in emotional regulation and school well-being. To analyze the predictive power of time attitude profiles on language and mathematics achievement, mixed-effects linear models with random intercepts at the classroom and educational institution levels were conducted, accounting for the nested structure of the data. Two separate analyses were performed for language and mathematics achievement, each controlling for age and gender as covariates. This preliminary model was conducted to assess the assumptions underlying the regression model on which the mediation analysis is based—assumptions that cannot be directly tested within the mediation model itself—as well as to evaluate potential multicollinearity issues among the variables and relevant covariables.

Finally, to examine the mediating role of emotion regulation and school well-being in academic achievement, two mediation models were estimated using structural equation modeling (SEM) with the lavaan package—one for language and one for mathematics—using the MLR estimator. In both models, paths were specified from time attitude profiles to multiple dimensions of emotion regulation and school well-being, and from these mediators to academic achievement. Indirect effects were evaluated using bootstrapped confidence intervals with 5000 resamples to obtain robust estimates. The models were estimated using unstandardized coefficients, as recommended in mediation analysis to preserve the metric of the variables and ensure accurate estimation of indirect effects (Hayes, 2018). Both models were saturated, including all correlations among exogenous and endogenous variables, as the primary objective was the estimation of indirect effects rather than model parsimony. Profile membership was dummy-coded into two variables (Positive and Optimistic), with the Ambivalent profile used as the reference category.

## 3. Results

### 3.1. Preliminary Analysis and Descriptive Statistics

Table 1 presents the descriptive statistics by sex for the study variables. Overall, the temporal profile suggests that females show less favorable indicators than males, with lower positive scores and higher negative scores, with effect sizes ranging from small (*d* = 0.22) for positive future to moderate (*d* = 0.70) for negative past. Likewise, females exhibit higher levels of emotion dysregulation, with moderate effect sizes ranging from *d* = 0.38 to *d* = 0.56. Finally, males show a slight advantage in subjective school well-being.

Table 2 presents the correlation matrix among the variables. The correlations among the personal profile variables range from moderate to strong. The relationships between profile variables and emotional dysregulation also fall within the weak to moderate range, with negative correlations for positive profile variables and positive correlations for negative profile variables. School satisfaction shows positive relationships with positive profile variables, and negative relationships with negative profile variables and emotional dysregulation. The performance variables show relationships ranging from negligible to small with the personal profile and dysregulation variables, with the strongest association observed with school satisfaction, at *r* = 0.26 for language and *r* = 0.25 for mathematics.

A Latent Profile Analysis (LPA) was conducted, resulting in the selection of a three-profile solution based on the lowest BIC value and theoretical parsimony. The optimal variance-covariance structure identified was ellipsoidal, with unequal volume but equal shape and orientation across profiles (VEE). This model yielded the following fit indices: BIC = 3696.25, AIC = 3543.47, SABIC = 3559.93, and an entropy of 0.718. Although the Bootstrap Sequential Likelihood Ratio Test (BLRT) indicated a significant difference only between the 1-profile and 2-profile solutions (*p* = 0.001), with non-significant results for the 2- vs. 3-profile (*p* = 0.570) and 3- vs. 4-profile (*p* = 0.053) comparisons, the three-profile solution was retained. This decision was driven by its greater theoretical richness, as it provided a more nuanced identification of cases with the most parsimonious indicators.

Table 3 presents the classification diagnostics for the three-profile solution. The model demonstrated adequate classification accuracy, as evidenced by the alignment between expected and observed class proportions. Classification quality was further supported by high mean a posteriori probability for each profile (APPM), with values of 0.91 for P1, 0.85 for P2, and 0.82 for P3. These values indicate that individuals were assigned to their respective profiles with a high degree of certainty and low cross-loading between groups.

**Figure 1:**
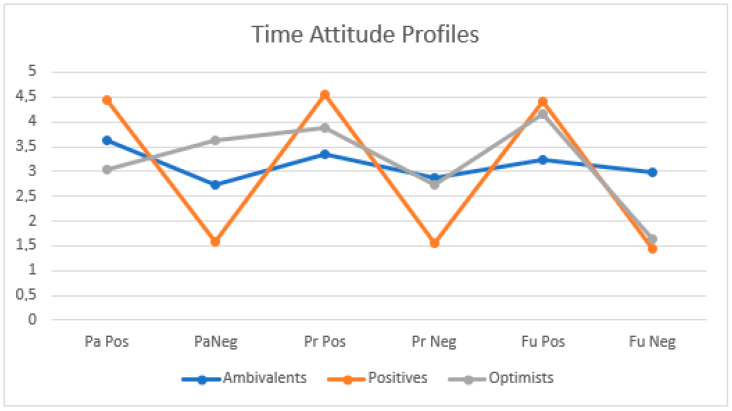
Time Attitude Profiles.

Three time attitude profiles were identified (see Figure 1): Ambivalents (*n* = 148), Positives (*n* = 54), and Optimists (*n* = 55). As can been seen in Table 4, mean time attitude scores differed meaningfully across the profiles (i.e., *g* > 0.41, Ferguson, 2009) for all but three of the 18 comparisons. The six time attitude scores for the Ambivalent profile were near the midpoint of the scale, generally falling between 2.5 and 3.5, indicating a moderate effect. Positives had high past, present, and future positive scores and low negative scores, reflecting globally positive affect toward the three time periods. Past and present time attitude scores for the Optimist profile were generally near the mid-point of the scales, but future positive and future negative scores for the Optimists were similar to the scores for the Positives; Optimists have moderate affect to the past and present but positive affect to the future.

Descriptive statistics were also calculated for school well-being, emotion regulation, and academic achievement by time attitude profile. These results are reported in Table 5. As can be seen, students with Positive profiles reported meaningfully lower emotional regulation difficulties on all five variables, as well as meaningfully higher levels of school well-being than Optimists and Ambivalents. Positives and Optimists also reported higher language and mathematics scores than Ambivalents, but these differences were neither statistically nor practically significant.

### 3.2. Mixed-Effects Linear Models

This model estimates the direct effects of time attitude profiles on academic achievement by including emotion regulation and school well-being as covariates. In this way, the mixed-effects models provide adjusted estimates of the direct effects, while also estimating the associations between the mediators and academic achievement (*b* paths), which are further examined within the mediation framework. Additionally, these models allow the assessment of key assumptions (e.g., linearity, normality, and homoscedasticity) within a multilevel structure. These models considered class, nested within school, as random effects (Table 6). It should be noted that low multicollinearity was observed among the predictor variables, with VIF < 1.52.

The model predicting language achievement was statistically significant, *F*(10, 442, 900) = 2.613, *p* = 0.004, with a Nakagawa pseudo-*R*^2^ of 0.089. However, the effect of time attitude profile was not significant when controlling for the other variables, *F*(2, 111,100) = 1.88, *p* = 0.153. It was observed that males showed lower average performance than females (*b* = −0.251, *p* = 0.013), and that higher levels of school well-being were associated with higher language performance (*b* = 0.121, *p* = 0.044). Upon examining the model assumptions, it was observed that the assumptions of linearity, normality, and homoscedasticity of residuals were met at both the individual and school levels.

The model predicting mathematics achievement was not statistically significant, *F*(10, 15, 832) = 1.819, *p* = 0.052, with a Nakagawa pseudo-*R*^2^ of 0.063. After adjusting for the remaining covariates, the time attitude profiles did not significantly predict outcomes, *F*(2, 2, 134) = 2.083, *p* = 0.125. It should be noted, however, that a statistically significant difference was observed, with higher mathematics achievement for the Optimistic profile compared to the Ambivalent profile when controlling for the remaining variables (*b* = 0.269, *p* = 0.047). The model assumptions were also met, including linearity, normality, and homoscedasticity of residuals at both the individual and school levels.

### 3.3. Mediation

To examine academic achievement using emotion regulation and school well-being as mediators, two mediation models were estimated via structural equation modeling—one for language and one for mathematics (Table 6). Sex was included as a covariate for academic achievement, whereas age was excluded as it did not show a significant effect in the mixed-effects models. Indirect effects were calculated as the product of the *a* paths (from time attitude profiles to emotion regulation and school well-being) and the *b* paths (from emotion regulation and school well-being to academic achievement). See Supplementary Material for full models.

Regarding the total effects of time attitude profiles on academic achievement, no significant differences were found between the Ambivalent profile and the Positive profile, nor between the Ambivalent and Optimistic profiles in language achievement. In mathematics, only the Optimistic profile showed higher achievement than the Ambivalent profile (*b* = 0.270, *p* = .047).

The effects of time attitudes profiles on emotion regulation and school well-being (*a* paths) were analyzed. The Positive profile showed significantly lower levels than the Ambivalent profile across all five emotion regulation dimensions, including emotional rejection (β = −1.09, *p* < .001), dyscontrol (β = −0.93, *p* < .001), interference (β = −0.71, *p* < .001), inattention (β = −0.80, *p* < .001), and confusion (β = −0.84, *p* < .001). In addition, the Positive profile significantly predicted higher levels of school well-being (β = 0.64, *p* < .001). The Optimistic profile did not differ significantly from the Ambivalent profile in most emotion regulation dimensions or school well-being, except for higher levels of emotional interference (β = 0.35, *p* = .018) and slightly lower emotional inattention (β = −0.27, *p* = .048).

In terms of the effects of school well-being and emotion regulation on academic achievement (*b* paths), school well-being was the only significant positive predictor in both language (β = 0.199, *p* = .001) and mathematics (β = 0.246, *p* < .001). Additionally, males scored significantly lower than females in language (β = −0.352, *p* = .001). No direct effects of time attitude profiles on academic achievement were statistically significant.

Regarding indirect effects, only two significant pathways were identified, both mediated by school well-being and associated with the Positive profile: one for mathematics achievement (ab = 0.16, 95% CI [0.07, 0.26]) and one for language achievement (ab = 0.131, 95% CI [0.049, 0.225]) (see Table 7).

## 4. Discussion

In this research, we aimed to determine whether the relationship between time attitude profiles and academic achievement is mediated by emotional regulation and school well-being. The results confirm that time attitude profiles are indirectly associated with academic achievement through school well-being. These findings parallel prior research indicating that adolescents maintaining favorable forward-looking orientations demonstrate superior academic performance. Mello and Worrell (2015) established this pattern empirically, showing that time perspective operates as a meaningful predictor of achievement outcomes across diverse adolescent samples. Contemporary investigations extend these initial observations, documenting that adolescents with well-developed future aspirations, coupled with constructive perspectives on their personal histories, consistently outperform peers who lack such temporal coherence (Hao et al., 2024).

The data demonstrate that students with a Positive time attitude profile experience higher levels of school well-being compared to those with Ambivalent time attitude profiles, whereas the Optimistic profile does not differ significantly from the Ambivalent time attitude profile in terms of perceived well-being. This pattern comports with theoretical models proposing that individuals who have higher positive and lower negative attitudes toward time report higher levels of psychological well-being and lower levels of psychological distress (Buhl & Lindner, 2009; Ranjbar et al., 2023). Conversely, characteristically rigid, extreme, or predominantly negative temporal perspectives precipitate psychological distress and reduced life satisfaction. Consistent with this framework, multiple investigations document that composite temporal orientations incorporating positive historical perspective, moderate present engagement, and aspiration-driven future focus constitute significant predictors of heightened subjective well-being in adolescent cohorts (Kuan & Zhang, 2022).

Furthermore, students with Positive time attitude profiles showed fewer emotion regulation difficulties than those with Ambivalent and Optimistic profiles. The Positive time attitude profile demonstrated significant adaptive effects on five emotion regulation dimensions, reducing difficulties in emotional rejection, dyscontrol, interference, and confusion. These empirical patterns align with emerging theoretical frameworks proposing that time perspective and affect regulation constitute interdependent processes. Adolescents whose time attitudes emphasize aversive historical experiences while minimizing future aspirations and investing primarily in immediate or fatalistic present concerns exhibit correspondingly elevated emotion regulation difficulties and diminished affective control capacity (Oyanadel et al., 2022).

However, in this study, neither for language nor mathematics did any emotion regulation dimension directly predict academic achievement. Empirical literature examining relationships between emotion regulation and academic achievement has generated heterogeneous findings; longitudinal investigations increasingly suggest that emotion regulation may exert indirect effects on achievement through multiple unmeasured or alternative mediating pathways (Wong et al., 2023). One plausible mechanism involves the possibility that emotion regulation primarily influences achievement-adjacent constructs such as academic motivation or classroom engagement, which subsequently influence achievement outcomes.

Along this line, empirical evidence identifies mediating mechanisms of different natures. On the one hand, from a well-being perspective, Seibert et al. (2017) proposed that school burnout acts as a bridge between emotion-regulation strategies and academic achievement, suggesting that stress management determines burnout levels, which in turn are associated with academic performance. Complementarily, from a cognitive–motivational standpoint, Usán and Quílez (2021) showed that self-efficacy significantly mediates this relationship, reinforcing the idea that emotional regulation operates through students’ perceived competence. This central role of self-efficacy has been confirmed by recent research, which highlights its mediating function in linking emotional factors to academic achievement; in this regard, it has been observed that this relationship is also enhanced by contextual variables such as social support (Wang & Wang, 2025). However, beyond the existence of these mediating mechanisms, the absence of a direct relationship in the present study might be explained by methodological factors. An alternative explanation suggests that the self-report measures used to assess emotional regulation may capture coping strategies that do not necessarily match those individuals actually display in everyday life; this underscores the need to use more sensitive and context-specific assessments (Koval et al., 2023). Although the scores on the scale used in this study (DERS-E) exhibited robust psychometric properties, it remains a measure of perceived difficulties; however, it has been argued that emotional regulation should be examined in a multifaceted and dynamic manner, integrating multiple levels of analysis (Sörman et al., 2022).

In contrast to the absence of direct effects of emotional regulation, the mediation analyses revealed explanatory mechanisms with greater predictive power within this model. Specifically, the hypothesis that school well-being acts as a mediator in the relationship between time-attitude profiles and academic performance was confirmed. These findings reflect a broader empirical consensus indicating that students who report lower school well-being show correspondingly compromised academic performance (Obermeier et al., 2021). Likewise, students’ satisfaction with schooling and their perceptions of the institutional climate are associated with higher grades (Daily et al., 2020). Research in Chilean educational contexts has specifically documented that students’ perceptions of social and academic integration within schools exert a substantial predictive influence on performance outcomes (Cerda et al., 2019), which reinforces the conclusion that school well-being operates as a significant determinant of school success.

In this way, the findings of the present study extend the emerging literature by documenting the mediating role of school well-being in the link between temporal-attitude profiles and academic performance. Although previous longitudinal-path studies have identified factors such as school satisfaction, enjoyment of learning, and academic self-confidence as variables that connect students’ individual differences with performance outcomes (Weber & Harzer, 2022), evidence on school well-being as a specific bridge remains scarce. Consequently, this study makes a meaningful contribution to current theoretical models by positioning school well-being as a key mechanism linking students’ temporal-attitude profiles to academic outcomes. These results not only reinforce the relevance of emotional variables in the school context but also underscore the need to consider well-being as a critical determinant of learning success.

### 4.1. Practical Implications

In light of the methodological design employed, these findings preliminarily suggest relevant implications for educational practice and the development of interventions. Schools seeking to improve academic outcomes could explore the incorporation of interventions that strengthen students’ positive time perspectives and enhance school well-being (Tejada-Gallardo et al., 2021). Following the framework proposed by Mello and Worrell (2015), such interventions might include activities that help students develop constructive attitudes toward time by facilitating reflection on positive past experiences, fostering realistic future aspirations, and cultivating a moderate engagement with present opportunities. In addition, it would be pertinent for schools to coordinate systemic efforts to improve the school climate, foster positive relationships with peers and teachers, and create a supportive academic environment. Given that the Positive profile was associated with the most favorable indicators in this sample, interventions aimed at nurturing positive perspectives on the past, positive orientations toward the present, and goal-oriented positive outlooks on the future may be particularly beneficial (Worrell et al., 2021; Worrell & Oyanadel, 2025).

### 4.2. Limitations and Future Directions

Several limitations should be noted. First, the cross-sectional design precludes drawing causal inferences; longitudinal studies would strengthen causal conclusions. Second, the sample was drawn from subsidized schools in specific regions of Chile, which may limit generalizability to other educational contexts or socioeconomic groups. Third, academic performance was assessed using school grades rather than standardized tests, which may vary across teachers and institutions. Fourth, the exclusive use of self-report measures and the inherent constraints of mediation models applied to cross-sectional data pose challenges for interpreting the scope of the model. Finally, potential moderators such as socioeconomic status and cultural background were not examined, even though they might influence the relationships under study.

Future research should employ longitudinal designs to examine reciprocal relationships between time perspective and academic outcomes, investigate the mechanisms through which emotional regulation may indirectly influence performance, and explore possible moderators of the observed effects. Moreover, given that this study was conducted in nine subsidized schools with similar socioeconomic profiles, future studies using broader samples of institutions with varying socioeconomic indices could adopt multilevel models to examine potential institutional effects. In addition, qualitative research exploring students’ subjective experiences of time, school satisfaction, and academic motivation could provide valuable insights for the development of interventions.

## 5. Conclusions

In conclusion, the findings of this study provide empirical support for the idea that school well-being is a relevant predictor of academic performance in key domains such as language and mathematics. Beyond its direct effect, the data indicate that school well-being operates as a mediating mechanism in the relationship between temporal-attitude profiles and academic performance.

One finding that invites theoretical reflection is the lack of predictive power of the emotional-regulation dimensions on academic performance. This result suggests that, although temporal-attitude profiles may foster greater emotional competence, the impact of these skills on academic achievement could be mediated by variables not captured in this model or that their influence is more subtle and requires more context-sensitive, situationally embedded assessments.

Furthermore, our results show that students with positive temporal-attitude profiles reported fewer difficulties in emotional regulation and higher levels of well-being compared to those with optimistic and ambivalent profiles. The empirical evidence presented here underscores the importance of temporal-attitude profiles as a potential intervention target in educational contexts. When conceptualized as a malleable individual-difference variable, it offers educators a promising pathway for promoting adolescents’ holistic development.

Based on these findings, educational institutions should consider implementing interventions aimed at strengthening temporal perspective and academic well-being as part of broader strategies to improve academic outcomes. Such interventions could include the explicit teaching of adaptive temporal-perspective strategies to foster a positive and balanced time perspective, activities designed to promote positive reflection on past experiences and future aspirations, and systemic efforts to enhance school climate and student–teacher relationships. By addressing temporal perspective and school well-being, educators can create conditions conducive to better academic performance and overall student well-being.

## Figures and Tables

**Table 1:** Comparison of variables by gender.

	Female		Male				
Variables	*M*	*SD*	*M*	*SD*	*t*-Test	*p*	*d*
Past Positive	3.686	0.901	3.959	0.932	t(565.9) = 3.55	<.001	0.30
Past Negative	2.998	1.037	2.296	0.978	t(559.6) = 8.29	<.001	0.70
Present Positive	3.585	0.929	3.998	0.846	t(554.7) = 5.54	<.001	0.47
Present Negative	2.771	0.958	2.228	0.914	t(560.8) = 6.91	<.001	0.58
Future Positive	3.569	0.986	3.784	0.923	t(558.6) = 2.67	.008	0.22
Future Negative	2.451	0.990	2.239	0.873	t(549.5) = 2.69	.007	0.23
Emotion Rejection	3.104	1.191	2.445	1.145	t(561.6) = 6.72	<.001	0.56
Emotional Dyscontrol	2.675	1.176	2.245	1.067	t(554.1) = 4.56	<.001	0.38
Emotional Interference	3.759	1.095	2.996	1.156	t(565.0) = 8.07	<.001	0.68
Lack of Emotional Attention	2.955	0.941	2.545	0.963	t(565.6) = 5.14	<.001	0.43
Emotional Confusion	3.245	1.062	2.607	1.144	t(565.7) = 6.90	<.001	0.58
School Satisfaction	4.389	0.820	4.547	0.861	t(562.0) = 2.23	.026	0.19

**Table 2:** Pearson’s correlations between variables.

	Npa	Ppr	Npr	PF	NF	ER	ED	Eint	Eina	EC	SWB	Lang	Mat
Positive past	−0.62 **	0.48 **	−0.35 **	0.22 **	−0.14 *	−0.33 **	−0.24 **	−0.25 **	−0.26 **	−0.19 **	0.25 **	0.01	0.1
Negative past	1 **	−0.33 **	0.53 **	−0.18 **	0.26 **	0.48 **	0.39 **	0.46 **	0.22 **	0.31 **	−0.18 **	−0.06	−0.12
Positive present		1 **	−0.66 **	0.47 **	−0.36 **	−0.36 **	−0.28 **	−0.26 **	−0.39 **	−0.27 **	0.38 **	0.13 *	0.23 **
Negative present			1 **	−0.32 **	0.39 **	0.49 **	0.4 **	0.38 **	0.29 **	0.35 **	−0.34 **	−0.16 *	−0.21 **
Positive future				1 **	−0.69 **	−0.16 *	−0.18 **	−0.14 *	−0.43 **	−0.16 **	0.35 **	0.06	0.06
Negative future					1 **	0.28 **	0.28 **	0.18 **	0.32 **	0.29 **	−0.24 **	−0.13 *	−0.16 *
Emotional rejection						1 **	0.59 **	0.64 **	0.18 **	0.55 **	−0.04	−0.02	−0.08
Emotional descontrol							1 **	0.6 **	0.19 **	0.43 **	−0.13 *	−0.13 *	−0.14 *
Emotional interference								1 **	0.17 **	0.49 **	−0.13 *	−0.07	−0.12
Emotional Inattention									1 **	0.23 **	−0.29 **	−0.12	−0.04
Emotional Confusion										1 **	−0.09	−0.02	−0.07
School Well-Being											1 **	0.26 **	0.25 **
Language												1 **	0.58 **

Note: * *p* < 0.05, ** *p* < 0.01. Npa = negative past; Ppr = positive present; Npr = negative present; PF = positive future; NF = negative future; ER = emotional rejection; ED = emotional dyscontrol; Eint = emotional interference; Eina = emotional inattention; EC = Emotional Confusion, SWB = school well-being; Lang = language achievement; Mat = mathematics achievement.

**Table 3:** Model fit indices by latent profiles.

	Probability		A Posteriori Probability Mean		
Profile	Expected	Observed	P1	P2	P3
P1: Ambivalents	0.575	0.574	0.91	0.03	0.06
P2: Positives	0.205	0.209	0.11	0.85	0.04
P3: Optimist	0.219	0.217	0.15	0.03	0.82

**Table 4:** Time Attitude Subscale Means by Time Perspective Profile.

Variable	Ambivalents (*n* = 148)	Positives (*n* = 54)	Optimists (*n* = 55)	*F*	*p*
Positive past	3.63 (0.83) ^a^	4.44 (0.50) ^b^	3.05 (0.89) ^c^	43.96	<.001
Negative past	2.73 (0.87) ^a^	1.58 (0.48) ^b^	3.63 (0.76) ^c^	95.91	<.001
Positive present	3.36 (0.87) ^a^	4.54 (0.51) ^b^	3.88 (0.70) ^c^	47.89	<.001
Negative present	2.86 (0.84) ^a^	1.56 (0.43) ^b^	2.73 (0.76) ^a^	61.39	<.001
Positive future	3.24 (0.92) ^b^	4.41 (0.60) ^a^	4.15 (0.72) ^a^	54.02	<.001
Negative future	2.98 (0.73) ^b^	1.45 (0.44) ^a^	1.65 (0.51) ^a^	162.5	<.001

Note: Values with different superscript letters reflect significant differences (*p* < .05), using the Tukey method for the three comparisons.

**Table 5:** Means and Standard Deviations of Outcome Variables by Time attitude profile.

Variable	Ambivalents (*n* = 148)	Positives (*n* = 54)	Optimists (*n* = 55)	*F*	*p*
Emotional rejection	3.01 (1.16) ^a^	1.92 (0.89) ^b^	3.16 (0.97) ^a^	24.49	<.001
Emotional dyscontrol	2.79 (1.1) ^a^	1.85 (1.01) ^b^	2.77(1.13) ^a^	15.2	<.001
Emotional interference	3.55 (1.14) ^a^	2.84 (1.21) ^b^	3.90 (0.87) ^a^	13.5	<.001
Emotional Inattention	3.02 (0.92) ^a^	2.23 (0.92) ^b^	2.75 (0.89) ^a^	14.89	<.001
Emotional Confusion	3.22 (1.11) ^a^	2.38 (0.93) ^b^	2.95 (1.03) ^a^	12.29	<.001
School Well-Being	4.23 (0.88) ^a^	4.86 (0.68) ^b^	4.36 (0.86) ^a^	10.55	<.001
Language	5.23 (0.81) ^a^	5.49 (0.83) ^a^	5.50 (0.79) ^a^	2.61	.07
Math	5.40 (0.90) ^a^	5.69 (0.82) ^a^	5.66 (0.87) ^a^	2.84	.06

Note: Values with different superscripts indicate significant differences (*p* < 0.05) using Tukey’s method for the three comparisons.

**Table 6:** Mixed-Effects Linear Models with Time Attitude Profiles and Academic Achievement in Language and Mathematics.

	Language Academic Achievement		Mathematics Academic Achievement	
Predictor	Estimate	*p*-Value	Estimate	*p*-Value
Intercept	7.013	<0.001	6.743	<0.001
Positive profile	0.044	0.743	0.132	0.376
Optimistic profile	0.227	0.052	0.269	0.047
Gender = Male	−0.251	0.013	0.114	0.304
Age	−0.128	0.166	−0.125	0.249
Emotional rejection	0.022	0.708	−0.015	0.821
Emotional dyscontrol	−0.094	0.083	−0.121	0.050
Emotional interference	−0.061	0.298	0.002	0.973
Emotional Inattention	−0.018	0.730	0.048	0.408
Emotional Confusion	0.047	0.369	0.032	0.580
School Well-Being	0.121	0.044	0.122	0.075

Note: The intercept corresponds to the ambivalent profile, female.

**Table 7:** Mediation Models with Time Attitude Profiles, Emotion Regulation, School Well-Being, and Achievement in Language and Mathematics.

	Language		Mathematics	
Predictor	Estimate (a × b)	CI (95%)	Estimate (a × b)	CI (95%)
Positive -> Rejection -> Grade	−0.032	−0.173, 0.099	0.004	−0.148, 0.153
Optimist -> Rejection -> Grade	0.005	−0.024, 0.044	−0.001	−0.039, 0.034
Positive -> dyscontrol -> Grade	0.069	−0.042, 0.190	0.066	−0.047, 0.188
Optimist -> dyscontrol -> Grade	0.002	−0.031, 0.041	0.002	−0.032, 0.041
Positive -> interference -> Grade	0.05	−0.044, 0.154	0.03	−0.070, 0.138
Optimist -> interference -> Grade	−0.025	−0.088, 0.020	−0.015	−0.074, 0.037
Positive -> inattention -> Grade	0.028	−0.073, 0.133	−0.054	−0.163, 0.045
Optimist -> inattention -> Grade	0.01	−0.028, 0.057	−0.018	−0.071, 0.017
Positive -> confusion -> Grade	−0.017	−0.114, 0.082	−0.012	−0.121, 0.096
Optimist -> confusion -> Grade	−0.005	−0.050, 0.032	−0.004	−0.053, 0.035
Positive -> School Well-Being -> Grade	**0.128**	**0.050, 0.224**	**0.16**	**0.071, 0.270**
Optimist -> School Well-Being -> Grade	0.027	−0.027, 0.092	0.034	−0.033, 0.113

Note: 95% confidence intervals based on 5000 bootstrap samples. Values in bold indicate statistically significant indirect effects.

## Data Availability

The datasets presented in this article are not readily available because of ethical restrictions.

## Supplementary Materials

The following supporting information can be downloaded at: https://www.mdpi.com/article/10.3390/jintelligence14060095/s1, Table S1: BIC by Number of Components and Model Type; Table S2: Complete SEM Model for Language Achievement; Table S3: Complete SEM Model for Mathematics Achievement.

## Abbreviations

The following abbreviations are used in this manuscript:

AATI-TA	Adolescent and Adult Time Inventory—Time Attitudes.
BASWBSS	Brief Subjective Well-Being Scale in School for Adolescents.
BIC	Bayesian Information Criterion.
BTP	Balanced Time Perspective.
CEBB	Comité de Ética y Bioética (Universidad de Concepción).
DERS-E	Difficulties in Emotion Regulation Scale (Spanish Version).
GPA	Grade Point Average.
LPA	Latent Profile Analysis.
SD	Standard Deviation.
SEM	Structural Equation Modeling
