# Supplementary material for: How Time Attitudes Shape Academic Success: The Protective Pathways of Emotion Regulation and School Well-Being in Adolescents
Source: J Intell. 2026 Jun 1;14(6):95. doi: 10.3390/jintelligence14060095 (PMC13301255; doi:10.3390/jintelligence14060095)
Supplement: Supplementary file 1 [file jintelligence-14-00095-s001.zip › jintelligence-4165348-supplementary.pdf]

Supplementary Material

Table S1: BIC by Number of Components and Model Type.

Groups	EII	VII	EEI	VEI	EVI	VVI	EEE	VEE	EVE	VVE	EEV	VEV	EVV	VVV
**3**	3902.3	3882.7	3924.0	3902.4	3926.5	3904.0	3714.5	**3696.3**	3742.2	3726.5	3816.2	3772.1	3856.8	3802.6
**4**	3865.2	3837.6	3891.1	3849.5	3902.2	3885.3	3722.2	3715.4	3755.8	3771.2	3862.3	3829.2	3926.7	3896.1
**5**	3853.8	3836.5	3882.5	3860.6	3950.7	3903.9	3751.5	3721.7	3787.1	3799.7	3961.9	3915.4	4056.1	4003.8
**6**	3856.1	3841.4	3874.4	3846.9	3977.5	3896.7	3776.1	3745.3	3829.7	3822.2	4042.6	3958.1	4143.6	4053.3
**7**	3870.5	3858.8	3891.2	3875.5	3985.4	3946.9	3797.7	3748.3	3876.7	3869.0	4043.5	4074.9	4198.5	4175.1
**8**	3893.2	3872.4	3903.0	3884.1	3989.4	3960.2	3830.4	3775.8	3929.5	3926.5	4120.8	4121.7	4319.9	4253.4
**9**	3884.5	3883.2	3913.1	3907.8	4018.1	3990.1	3847.5	3817.9	3965.9	3935.7	4211.4	4225.4	4372.5	4303.0

Note: The 14 models for covariance matrix proposed by Banfield and Raftery (1993) were evaluated for models with 3 to 9 clusters. The best model (in bold) was selected based on the lowest BIC value, which balances goodness-of-fit with model complexity. The abbreviations below describe the properties of the covariance matrix for each model: EII: Spherical, equal volume. VII: Spherical, unequal volume. EEI: Diagonal, equal volume and shape. VEI: Diagonal, unequal volume, equal shape. EVI: Diagonal, equal volume, unequal shape. VVI: Diagonal, unequal volume and shape. EEE: Ellipsoidal, equal volume, shape, and orientation. VEE: Ellipsoidal, unequal volume, equal shape and orientation. EVE: Ellipsoidal, equal volume and orientation, unequal shape. VVE: Ellipsoidal, unequal volume and orientation, equal shape. EEV: Ellipsoidal, equal volume and shape, unequal orientation. VEV: Ellipsoidal, unequal volume and shape, equal orientation. EVV: Ellipsoidal, equal volume, unequal shape and orientation. VVV: Ellipsoidal, unequal volume, shape, and orientation.

Table S2: Complete SEM Model for Language Achievement.

lhs	op	rhs	label	est	se	z	pvalue	CI
n.leng.t1	~	ders.rech.t1	b1	0.029	0.063	0.465	0.642	-0.091 , 0.153
n.leng.t1	~	ders.desc.t1	b2	-0.074	0.061	-1.23	0.221	-0.194 , 0.044
n.leng.t1	~	ders.int.t1	b3	-0.071	0.066	-1.07	0.285	-0.200 , 0.057
n.leng.t1	~	ders.des.t1	b4	-0.035	0.063	-0.554	0.58	-0.157 , 0.088
n.leng.t1	~	ders.conf.t1	b5	0.02	0.059	0.35	0.727	-0.095 , 0.132
**n.leng.t1**	**~**	**bas.sat.t1**	**b6**	**0.199**	**0.061**	**3.25**	**0.001**	**0.082 , 0.323**
n.leng.t1	~	es.positivo	c1	0.079	0.147	0.538	0.59	-0.215 , 0.359
n.leng.t1	~	es.optimista	c2	0.204	0.123	1.66	0.096	-0.041 , 0.437
**n.leng.t1**	**~**	**es.hombre**		**-0.352**	**0.103**	**-3.43**	**0.001**	**-0.553 , -0.145**
**ders.rech.t1**	**~**	**es.positivo**	**a11**	**-1.09**	**0.155**	**-7.02**	**0**	**-1.391 , -0.779**
ders.rech.t1	~	es.optimista	a12	0.178	0.164	1.08	0.278	-0.137 , 0.494
**ders.desc.t1**	**~**	**es.positivo**	**a21**	**-0.932**	**0.164**	**-5.7**	**0**	**-1.246 , -0.596**
ders.desc.t1	~	es.optimista	a22	-0.028	0.176	-0.161	0.872	-0.377 , 0.318
**ders.int.t1**	**~**	**es.positivo**	**a31**	**-0.713**	**0.187**	**-3.82**	**0**	**-1.077 , -0.337**
ders.int.t1	~	es.optimista	a32	0.349	0.148	2.36	**0.018**	0.060 , 0.638

lhs	op	rhs	label	est	se	z	pvalue	CI
**ders.des.t1**	**~**	**es.positivo**	**a41**	**-0.795**	**0.145**	**-5.47**	**0**	**-1.073 , -0.496**
ders.des.t1	~	es.optimista	a42	-0.274	0.138	-1.98	**0.048**	-0.545 , -0.005
**ders.conf.t1**	**~**	**es.positivo**	**a51**	**-0.84**	**0.154**	**-5.46**	**0**	**-1.140 , -0.538**
ders.conf.t1	~	es.optimista	a52	-0.268	0.165	-1.62	0.106	-0.595 , 0.058
**bas.sat.t1**	**~**	**es.positivo**	**a61**	**0.644**	**0.117**	**5.49**	**0**	**0.413 , 0.874**
bas.sat.t1	~	es.optimista	a62	0.137	0.136	1.01	0.313	-0.137 , 0.402
ders.rech.t1	~~	es.hombre		-0.138	0.031	-4.42	0	-0.199 , -0.076
ders.desc.t1	~~	es.hombre		-0.078	0.033	-2.36	0.018	-0.140 , -0.013
ders.int.t1	~~	es.hombre		-0.191	0.032	-6	0	-0.251 , -0.125
ders.des.t1	~~	es.hombre		-0.035	0.028	-1.25	0.21	-0.088 , 0.021
ders.conf.t1	~~	es.hombre		-0.143	0.032	-4.54	0	-0.204 , -0.079
bas.sat.t1	~~	es.hombre		-0.002	0.026	-0.069	0.945	-0.052 , 0.051
es.positivo	~~	es.hombre		0.022	0.013	1.78	0.075	-0.003 , 0.047
es.optimista	~~	es.hombre		-0.032	0.013	-2.52	0.012	-0.057 , -0.007
es.positivo	~~	es.optimista		-0.045	0.007	-6.9	0	-0.059 , -0.033
ders.rech.t1	~~	ders.desc.t1		0.619	0.075	8.25	0	0.465 , 0.761
ders.rech.t1	~~	ders.int.t1		0.692	0.071	9.75	0	0.547 , 0.825
ders.rech.t1	~~	ders.des.t1		0.078	0.062	1.26	0.208	-0.044 , 0.197
ders.rech.t1	~~	ders.conf.t1		0.567	0.073	7.76	0	0.417 , 0.711
ders.rech.t1	~~	bas.sat.t1		0.065	0.059	1.09	0.277	-0.052 , 0.182
ders.desc.t1	~~	ders.int.t1		0.672	0.075	8.91	0	0.519 , 0.811
ders.desc.t1	~~	ders.des.t1		0.104	0.07	1.49	0.136	-0.034 , 0.239
ders.desc.t1	~~	ders.conf.t1		0.428	0.078	5.52	0	0.274 , 0.578
ders.desc.t1	~~	bas.sat.t1		-0.044	0.058	-0.758	0.448	-0.157 , 0.071
ders.int.t1	~~	ders.des.t1		0.103	0.066	1.56	0.118	-0.025 , 0.232
ders.int.t1	~~	ders.conf.t1		0.539	0.075	7.18	0	0.388 , 0.682
ders.int.t1	~~	bas.sat.t1		-0.056	0.052	-1.07	0.283	-0.160 , 0.046
ders.des.t1	~~	ders.conf.t1		0.141	0.071	2	0.046	-0.001 , 0.277
ders.des.t1	~~	bas.sat.t1		-0.165	0.047	-3.55	0	-0.257 , -0.074
ders.conf.t1	~~	bas.sat.t1		-0.001	0.057	-0.011	0.991	-0.115 , 0.113
n.leng.t1	~~	n.leng.t1		0.579	0.044	13.1	0	0.470 , 0.645
ders.rech.t1	~~	ders.rech.t1		1.14	0.077	14.8	0	0.983 , 1.281
ders.desc.t1	~~	ders.desc.t1		1.17	0.087	13.4	0	0.997 , 1.337
ders.int.t1	~~	ders.int.t1		1.2	0.08	15	0	1.029 , 1.346
ders.des.t1	~~	ders.des.t1		0.83	0.066	12.6	0	0.692 , 0.953
ders.conf.t1	~~	ders.conf.t1		1.11	0.071	15.7	0	0.961 , 1.243
bas.sat.t1	~~	bas.sat.t1		0.691	0.063	11	0	0.564 , 0.807
es.positivo	~~	es.positivo		0.165	0.014	11.4	0	0.136 , 0.192
es.optimista	~~	es.optimista		0.17	0.014	11.8	0	0.139 , 0.196
es.hombre	~~	es.hombre		0.25	0.001	177	0	0.245 , 0.250
n.leng.t1	~1			4.97	0.404	12.3	0	4.199 , 5.751
ders.rech.t1	~1			3.01	0.095	31.7	0	2.826 , 3.195

lhs	op	rhs	label	est	se	z	pvalue	CI
ders.desc.t1	~1			2.79	0.089	31.3	0	2.610 , 2.963
ders.int.t1	~1			3.55	0.093	38.1	0	3.366 , 3.730
ders.des.t1	~1			3.02	0.074	40.9	0	2.876 , 3.166
ders.conf.t1	~1			3.22	0.092	35.2	0	3.045 , 3.406
bas.sat.t1	~1			4.23	0.072	58.5	0	4.088 , 4.367
es.positivo	~1			0.209	0.025	8.36	0	0.163 , 0.260
es.optimista	~1			0.217	0.026	8.49	0	0.167 , 0.267
es.hombre	~1			0.504	0.031	16.1	0	0.442 , 0.566
ab.11	:=	a11*b1	ab.11	-0.032	0.069	-0.458	0.647	-0.173 , 0.099
ab.12	:=	a12*b1	ab.12	0.005	0.016	0.322	0.748	-0.024 , 0.044
ab.21	:=	a21*b2	ab.21	0.069	0.058	1.19	0.233	-0.042 , 0.190
ab.22	:=	a22*b2	ab.22	0.002	0.017	0.125	0.9	-0.031 , 0.041
ab.31	:=	a31*b3	ab.31	0.05	0.049	1.02	0.308	-0.044 , 0.154
ab.32	:=	a32*b3	ab.32	-0.025	0.027	-0.91	0.363	-0.088 , 0.020
ab.41	:=	a41*b4	ab.41	0.028	0.051	0.544	0.586	-0.073 , 0.133
ab.42	:=	a42*b4	ab.42	0.01	0.02	0.48	0.631	-0.028 , 0.057
ab.51	:=	a51*b5	ab.51	-0.017	0.05	-0.344	0.731	-0.114 , 0.082
ab.52	:=	a52*b5	ab.52	-0.005	0.019	-0.285	0.776	-0.050 , 0.032
ab.61	:=	a61*b6	ab.61	0.128	0.044	2.89	0.004	0.050 , 0.224
ab.62	:=	a62*b6	ab.62	0.027	0.03	0.924	0.355	-0.027 , 0.092

Table S3: Complete SEM Model for Mathematics Achievement.

lhs	op	rhs	label	est	se	z	pvalue	ic
n.mat.t1	~	ders.rech.t1	b1	-0.003	0.068	-0.05	0.96	-0.137 , 0.133
n.mat.t1	~	ders.desc.t1	b2	-0.07	0.061	-1.15	0.249	-0.189 , 0.049
n.mat.t1	~	ders.int.t1	b3	-0.043	0.07	-0.611	0.541	-0.179 , 0.092
n.mat.t1	~	ders.des.t1	b4	0.067	0.062	1.08	0.28	-0.057 , 0.191
n.mat.t1	~	ders.conf.t1	b5	0.014	0.064	0.217	0.828	-0.112 , 0.140
**n.mat.t1**	**~**	**bas.sat.t1**	**b6**	**0.246**	**0.067**	**3.67**	**0**	**0.118 , 0.384**
n.mat.t1	~	es.positivo	c1	0.073	0.153	0.479	0.632	-0.221 , 0.383
n.mat.t1	~	es.optimista	c2	0.276	0.145	1.91	0.057	-0.009 , 0.563
n.mat.t1	~	es.hombre		0.071	0.112	0.628	0.53	-0.142 , 0.294
**ders.rech.t1**	**~**	**es.positivo**	**a11**	**-1.09**	**0.155**	**-7.02**	**0**	**-1.391 , -0.779**
ders.rech.t1	~	es.optimista	a12	0.178	0.164	1.08	0.278	-0.137 , 0.494
**ders.desc.t1**	**~**	**es.positivo**	**a21**	**-0.932**	**0.164**	**-5.7**	**0**	**-1.246 , -0.596**
ders.desc.t1	~	es.optimista	a22	-0.028	0.176	-0.161	0.872	-0.377 , 0.318
**ders.int.t1**	**~**	**es.positivo**	**a31**	**-0.713**	**0.187**	**-3.82**	**0**	**-1.077 , -0.337**
**ders.int.t1**	**~**	**es.optimista**	**a32**	**0.35**	**0.148**	**2.36**	**0.018**	**0.062 , 0.639**
**ders.des.t1**	**~**	**es.positivo**	**a41**	**-0.795**	**0.145**	**-5.47**	**0**	**-1.073 , -0.496**
**ders.des.t1**	**~**	**es.optimista**	**a42**	**-0.274**	**0.138**	**-1.98**	**0.048**	**-0.545 , -0.005**

lhs	op	rhs	label	est	se	z	pvalue	ic
**ders.conf.t1**	**~**	**es.positivo**	**a51**	**-0.84**	**0.154**	**-5.46**	**0**	**-1.140 , -0.538**
ders.conf.t1	~	es.optimista	a52	-0.268	0.165	-1.62	0.106	-0.595 , 0.058
**bas.sat.t1**	**~**	**es.positivo**	**a61**	**0.649**	**0.117**	**5.54**	**0**	**0.420 , 0.878**
bas.sat.t1	~	es.optimista	a62	0.137	0.136	1.01	0.313	-0.137 , 0.402
ders.rech.t1	~~	es.hombre		-0.138	0.031	-4.42	0	-0.199 , -0.076
ders.desc.t1	~~	es.hombre		-0.078	0.033	-2.36	0.018	-0.140 , -0.013
ders.int.t1	~~	es.hombre		-0.191	0.032	-6	0	-0.251 , -0.125
ders.des.t1	~~	es.hombre		-0.035	0.028	-1.25	0.21	-0.088 , 0.021
ders.conf.t1	~~	es.hombre		-0.143	0.032	-4.54	0	-0.204 , -0.079
bas.sat.t1	~~	es.hombre		-0.001	0.026	-0.052	0.958	-0.052 , 0.051
es.positivo	~~	es.hombre		0.022	0.013	1.78	0.075	-0.003 , 0.047
es.optimista	~~	es.hombre		-0.032	0.013	-2.52	0.012	-0.057 , -0.007
es.positivo	~~	es.optimista		-0.045	0.007	-6.9	0	-0.059 , -0.033
ders.rech.t1	~~	ders.desc.t1		0.619	0.075	8.25	0	0.465 , 0.761
ders.rech.t1	~~	ders.int.t1		0.692	0.071	9.76	0	0.547 , 0.825
ders.rech.t1	~~	ders.des.t1		0.078	0.062	1.26	0.208	-0.044 , 0.197
ders.rech.t1	~~	ders.conf.t1		0.567	0.073	7.76	0	0.417 , 0.711
ders.rech.t1	~~	bas.sat.t1		0.065	0.059	1.09	0.276	-0.051 , 0.182
ders.desc.t1	~~	ders.int.t1		0.671	0.075	8.91	0	0.518 , 0.811
ders.desc.t1	~~	ders.des.t1		0.104	0.07	1.49	0.136	-0.034 , 0.239
ders.desc.t1	~~	ders.conf.t1		0.428	0.078	5.52	0	0.274 , 0.578
ders.desc.t1	~~	bas.sat.t1		-0.044	0.058	-0.766	0.444	-0.158 , 0.070
ders.int.t1	~~	ders.des.t1		0.103	0.066	1.56	0.118	-0.025 , 0.232
ders.int.t1	~~	ders.conf.t1		0.539	0.075	7.18	0	0.388 , 0.682
ders.int.t1	~~	bas.sat.t1		-0.056	0.052	-1.07	0.285	-0.160 , 0.046
ders.des.t1	~~	ders.conf.t1		0.141	0.071	2	0.046	-0.001 , 0.277
ders.des.t1	~~	bas.sat.t1		-0.166	0.047	-3.56	0	-0.258 , -0.075
ders.conf.t1	~~	bas.sat.t1		-0.001	0.058	-0.025	0.98	-0.116 , 0.113
n.mat.t1	~~	n.mat.t1		0.683	0.061	11.2	0	0.541 , 0.779
ders.rech.t1	~~	ders.rech.t1		1.14	0.077	14.8	0	0.983 , 1.281
ders.desc.t1	~~	ders.desc.t1		1.17	0.087	13.4	0	0.997 , 1.337
ders.int.t1	~~	ders.int.t1		1.2	0.08	15	0	1.029 , 1.346
ders.des.t1	~~	ders.des.t1		0.83	0.066	12.6	0	0.692 , 0.953
ders.conf.t1	~~	ders.conf.t1		1.11	0.071	15.7	0	0.961 , 1.243
bas.sat.t1	~~	bas.sat.t1		0.691	0.063	11	0	0.565 , 0.807
es.positivo	~~	es.positivo		0.165	0.014	11.4	0	0.136 , 0.192
es.optimista	~~	es.optimista		0.17	0.014	11.8	0	0.139 , 0.196
es.hombre	~~	es.hombre		0.25	0.001	177	0	0.245 , 0.250
n.mat.t1	~1			4.46	0.427	10.4	0	3.604 , 5.247
ders.rech.t1	~1			3.01	0.095	31.7	0	2.826 , 3.195
ders.desc.t1	~1			2.79	0.089	31.3	0	2.610 , 2.963
ders.int.t1	~1			3.55	0.093	38.1	0	3.366 , 3.730

lhs	op	rhs	label	est	se	z	pvalue	ic
ders.des.t1	~1			3.02	0.074	40.9	0	2.876 , 3.166
ders.conf.t1	~1			3.22	0.092	35.2	0	3.045 , 3.406
bas.sat.t1	~1			4.23	0.072	58.5	0	4.088 , 4.367
es.positivo	~1			0.209	0.025	8.36	0	0.163 , 0.260
es.optimista	~1			0.217	0.026	8.49	0	0.167 , 0.267
es.hombre	~1			0.504	0.031	16.1	0	0.442 , 0.566
ab.11	:=	a11*b1	ab.11	0.004	0.075	0.049	0.961	-0.148 , 0.153
ab.12	:=	a12*b1	ab.12	-0.001	0.017	-0.035	0.972	-0.039 , 0.034
ab.21	:=	a21*b2	ab.21	0.066	0.059	1.11	0.268	-0.047 , 0.188
ab.22	:=	a22*b2	ab.22	0.002	0.017	0.119	0.906	-0.032 , 0.041
ab.31	:=	a31*b3	ab.31	0.03	0.051	0.591	0.555	-0.070 , 0.138
ab.32	:=	a32*b3	ab.32	-0.015	0.027	-0.548	0.584	-0.074 , 0.037
ab.41	:=	a41*b4	ab.41	-0.054	0.052	-1.03	0.305	-0.163 , 0.045
ab.42	:=	a42*b4	ab.42	-0.018	0.022	-0.845	0.398	-0.071 , 0.017
ab.51	:=	a51*b5	ab.51	-0.012	0.054	-0.215	0.83	-0.121 , 0.096
ab.52	:=	a52*b5	ab.52	-0.004	0.021	-0.18	0.858	-0.053 , 0.035
ab.61	:=	a61*b6	ab.61	0.16	0.05	3.17	0.002	0.071 , 0.270
ab.62	:=	a62*b6	ab.62	0.034	0.037	0.924	0.356	-0.033 , 0.113
